# Biphasic Electrical Currents Stimulation Promotes both Proliferation and Differentiation of Fetal Neural Stem Cells

**DOI:** 10.1371/journal.pone.0018738

**Published:** 2011-04-13

**Authors:** Keun-A Chang, Jin Won Kim, Jeong a Kim, Sungeun Lee, Saeromi Kim, Won Hyuk Suh, Hye-Sun Kim, Sunghoon Kwon, Sung June Kim, Yoo-Hun Suh

**Affiliations:** 1 Department of Pharmacology, College of Medicine, Neuroscience Research Institute, MRC, Seoul National University, Seoul, South Korea; 2 Department of Pharmacology, Bundang Hospital, College of Medicine, Seoul National University, Bundang-Gu, Sungnam, Kyungki, South Korea; 3 School of Electrical Engineering and Computer Science, Seoul National University, Seoul, Republic of Korea; 4 Department of Bioengineering, University of California, Berkeley, California, United States of America; University of Sao Paulo - USP, Brazil

## Abstract

The use of non-chemical methods to differentiate stem cells has attracted researchers from multiple disciplines, including the engineering and the biomedical fields. No doubt, growth factor based methods are still the most dominant of achieving some level of proliferation and differentiation control - however, chemical based methods are still limited by the quality, source, and amount of the utilized reagents. Well-defined non-chemical methods to differentiate stem cells allow stem cell scientists to control stem cell biology by precisely administering the pre-defined parameters, whether they are structural cues, substrate stiffness, or in the form of current flow. We have developed a culture system that allows normal stem cell growth and the option of applying continuous and defined levels of electric current to alter the cell biology of growing cells. This biphasic current stimulator chip employing ITO electrodes generates both positive and negative currents in the same culture chamber without affecting surface chemistry. We found that biphasic electrical currents (BECs) significantly increased the proliferation of fetal neural stem cells (NSCs). Furthermore, BECs also promoted the differentiation of fetal NSCs into neuronal cells, as assessed using immunocytochemistry. Our results clearly show that BECs promote both the proliferation and neuronal differentiation of fetal NSCs. It may apply to the development of strategies that employ NSCs in the treatment of various neurodegenerative diseases, such as Alzheimer's and Parkinson's diseases.

## Introduction

Neural stem cells (NSCs) are self-renewing cells that maintain the capability to differentiate into the major cell types of the brain. Several groups have consistently demonstrated that NSCs in the developing and adult brain generate mature cells of all neural lineages, including astrocytes, oligodendrocytes, and neurons ([1], [2], [3]). Embryonic central nervous tissue derived NSCs can spontaneously (after factor removal) differentiated into astrocytes, oligodendrocytes, and neurons in an approximate ratio of 25∶5∶1, respectively. ([2], [4]).

NSCs are being considered for important therapeutic strategies for the treatment of various neurodegenerative diseases including Alzheimer's and Parkinson's diseases. NSCs taken from human fetuses have shown a remarkable ability to replace endogenous, degenerating dopamine neurons and to ameliorate disease symptoms [4]. It has already been shown that hippocampus-derived NSCs can differentiate into excitatory and inhibitory neurons expressing the appropriate neurotransmitters [5] and/or form functional synapses with co-cultured neurons or within the mouse brain [6].

For clinical therapy using NSCs, it is essential to secure a large number of differentiated NSCs. The transplantation of differentiated NSCs would enhance engraftment efficiency and clinical efficiency [7]. Although much work has been done to elucidate the regulatory mechanisms that control the proliferation and differentiation of endogenous NSCs, this topic requires further investigation. The manipulation of central nervous system (CNS) stem cells *in vitro* could be useful for understanding of the mechanisms that control the proliferation and fate choice of NSCs. Fetal and adult stem cells can adopt neuronal and glial fates *in vitro* ([8], [9], [10], [11]) in response to signals that control the early steps in fate choice [10]. Previous studies have shown the regulatory effects of growth factors on the differentiation of CNS precursor cells into different types of neurons ([5], [12], [13], [14], [15]). In addition, multiple stages of neurogenesis, including progenitor proliferation, neural differentiation, and neural maturation, have been shown to be regulated by neural activity ([16], [17]).

**Figure 1:**
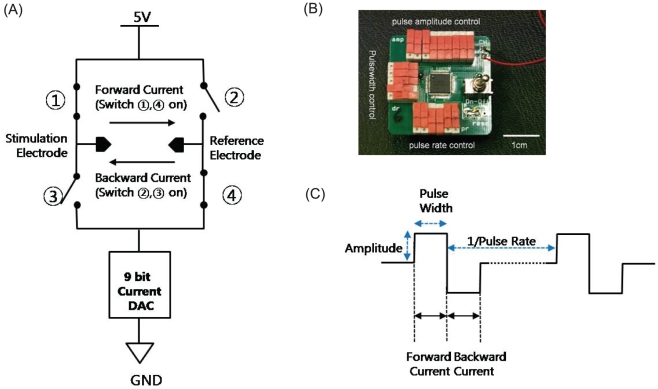
Biphasic electrical current stimulator. (A) A circuit diagram: Biphasic current is generated by the complementary switching between 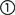, 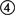 and 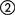, 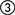 switches. (B) A top view including the on/off switches for amplitude, duration, and pulse rate setting. (C) Current pulses.

**Figure 2:**
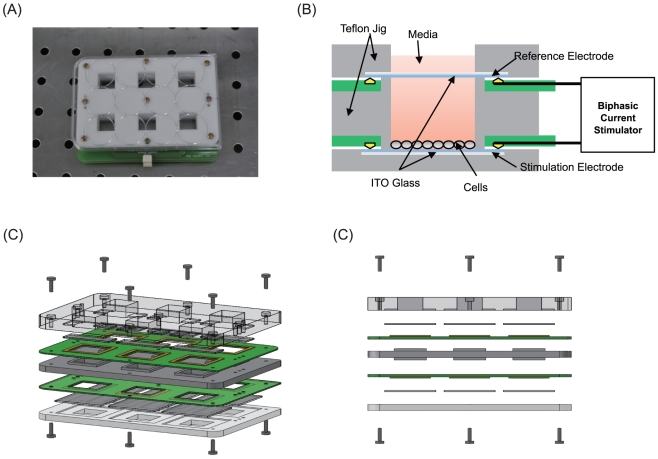
The schematic diagrams of the Teflon culture dish. The Teflon® culture dish and the ITO-deposited glass plates for culturing the neural stem cells are connected to a biphasic current stimulator. Each well is connected in a shunt electrical configuration, forming a 6-well culture system. (A) Photo of the culture chamber. (B) Schematic diagram of cross section of the culture chamber. (C) 3D view of the setup. (D) Side view of the setup.

In this study, we engineered a biphasic electrical current stimulator chip (Figure 1) that modulates electrical fields on living cells to investigate the effects of electric fields on fetal NSC proliferation and differentiation processes. Freshly isolated NSCs were cultured in a custom-made electrostimulation culture system (Figure 2), which involved the generation of both positive and negative currents inside the six individual culture chambers without detrimental effects and heterogeneity. It is known that the physiological function of *in vitro* cultured cells could be dramatically altered in response to physical stimuli including electromagnetic field, mechanical force and heat treatment [7]. Charge-balanced BEC is coupled with changes in an electromagnetic field. It can also avoid cell damage by charge accumulation and decrease of stimulation efficiency by charged protein accumulation. The BEC culture system surface was designed to incorporate a conductive and optically transparent layer of indium tin oxide (ITO) that facilitated both optical observation and direct electrical stimulation in liquid phase at 37 °C and 5% CO_2_. The custom designed biphasic electrical current stimulator chip (Figure 1) allowed easy access to control the duration and amplitude of electric currents being applied to the NSCs. We hypothesized that NSC proliferation and differentiation will be affected differently by how the electrical currents are modulated.

## Results

### Biphasic electrical current stimulator chip and *In Vitro* culture system

The waveforms of the fabricated BEC stimulator chip were tested. Measured amplitudes and durations showed good linear characteristic against set levels. Although the waveforms of positive and negative phases are not completely symmetric, the DC potential by charge accumulation was not observed. We believe that, during the culture period, the accumulated charges will have discharged by electrical short circuit thus not significantly affecting cell proliferation. In general, continuous BEC stimulation without short circuit generates a constant DC potential by charges accumulated at the parasitic capacitance of the electrode-electrolyte interface. Power consumption of a BEC stimulator chip was ideal enough to maintain a stable BEC stimulation for 7 days of culturing inside the incubator.

The *In vitro* BEC culture system (Figure 2), composed of Indium Tin Oxide (ITO) glasses, Teflon jigs, printed circuit boards (PCBs) and elastomer O-rings, did not induce conspicuous cell death. The elatomer O-rings completely blocked the leakage of culture media, and PCBs that can influence cell death were isolated from NSCs and culture media. ITO covered glass was transparent enough to observe the morphology and growth of cells under stimulation during the culture period. In addition, the impedance values of ITO glasses were so uniform that BEC from a stimulator chip was distributed equally to all six-culture chambers.

### Electrical stimulation by BEC increases the proliferation of fetal NSCs

**Figure 3:**
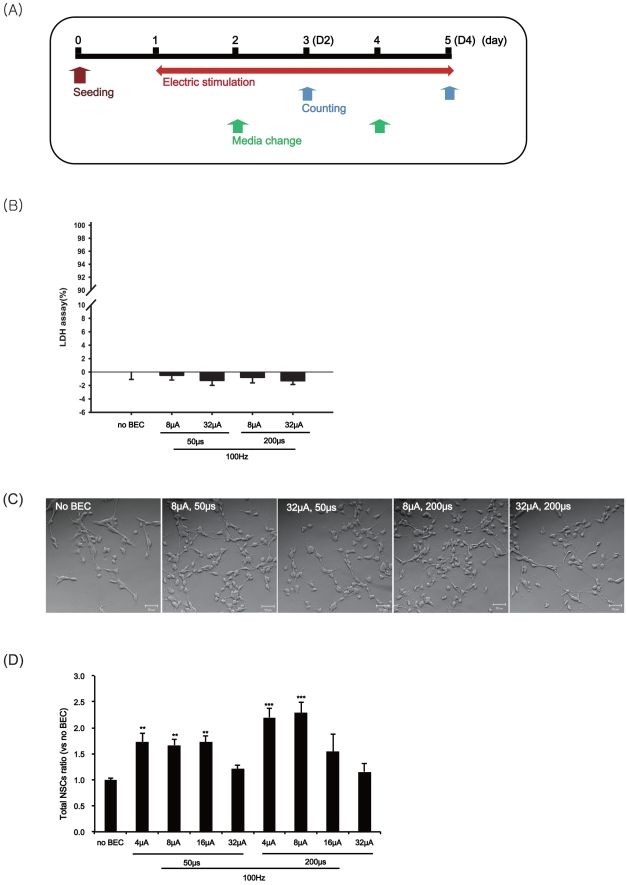
The effects of BEC on the proliferation of fetal NSCs. The stem cells were stimulated at 100 Hz with a magnitude of 4, 8, 16 or 32 µA/cm^2^ and duration of 50 or 200 µs for 4 days and then counted the numbers of stem cells. (A) Experimental scheme with the electrical stimulation during the proliferation. (B) Cytotoxicity of BEC in fetal NSCs using LDH assay. Values are expressed as mean ± SEM of five to eight independent experiments. Data bars are not significantly different from No BEC group by ANOVA with Turkey test. (C) A representative phase contrast micrograph of fetal NSCs produced with each condition of BEC for 4 days; No BEC, 8 µA/cm^2^ for 50 µs, 8 µA/cm^2^ for 200 µs, 32 µA/cm^2^ for 50 µs, 32 µA/cm^2^ for 200 µs. (D) After 4 days of the electrical stimulation, cell numbers were counted with trypan blue staining. Data represent mean ± S.E.M. of *n* = 4–17. **, *p*≤0.01, ***, *p*≤0.001; by One-Way ANOVA: Tukey's HSD Post Hoc test.

In this study, we investigated the effects of electrical stimulation by BEC on the proliferation of NSCs using a biphasic electrical current stimulator chip under several different amplitude and duration settings. The experimental scheme for the proliferation assessment is shown in Figure 3A. We initially cultured NSCs to assess proliferation. NSCs were cultured as single adherent cells on laminin-coated surfaces or as the neurospheres and the numbers were counted. In the case of single adherent cell cultures (P3; fetal NSCs after three passages), we investigated the four-day effects of eight different BEC conditions and exposed the fetal NSCs to a 100 Hz electrical stimulation with a magnitude of 4, 8, 16 and 32 µA/cm^2^ with 50 and 200 µs pulses in a continuous manner (Figure 3). We verified that four different electric current conditions did not particularly exert neurotoxicity on fetal NSCs (Figure 3B). The NSCs showed normal adherent cell-like behavior over the laminin-coated ITO surfaces with or without the four different BEC settings (Figure 3C). A more wider range study revealed that the total number of neural stem cells increased, specially using a magnitude ofif the magnitude was set to 8 µA/cm^2^ withfor 200 µs pulses (100 Hz), the total number of NSC count was more than double of controls (ratio = 2.29 (±0.21) (Figure 3D).

**Figure 4:**
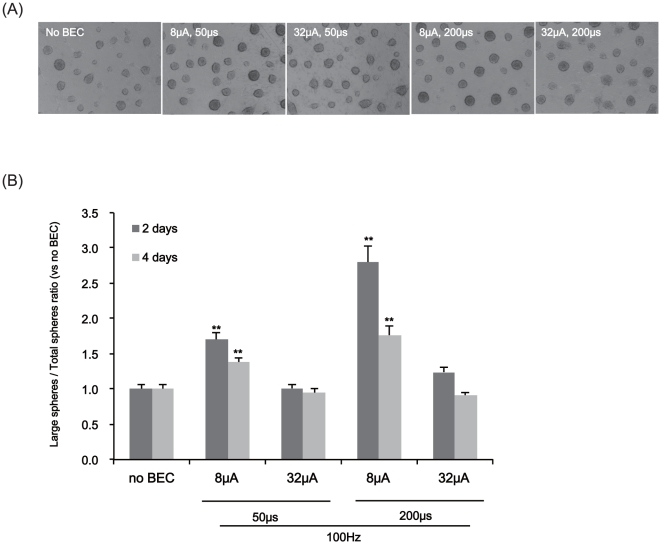
The effects of BEC on the production of neurospheres. For the neurosphere assay, we stimulated them at 100 Hz with a magnitude of 8 or 32 µA/cm^2^ and duration of 50 or 200 µs for 2 and 4 days and then counted the numbers of neurospheres after 2 and 4 days of electrical stimulation. (A) A representative phase contrast micrograph of neurospheres produced with four conditions of BEC for 4 days. (B) Ratios of neurospheres greater than 100 µm in diameter (large spheres) versus total neurospheres of all sizes (total spheres) after 2 and 4 days of electrical stimulation. Data represent mean ± S.E.M. of *n* = 37–52. **, *p*≤0.01, ***, *p*≤0.001; by One-Way ANOVA: Tukey's HSD Post Hoc test.

For the neurosphere assay (Figure 4), neurospheres of fetal NSCs were exposed to a 100 Hz electrical stimulation with a magnitude of 8 and 32 µA/cm^2^ with 50 or 200 µs pulses in a continuous mode during their proliferation phase (4 days) after three passages (P3). Under the light microscope, we counted both the total number of neurospheres and the number of large neurospheres over 100 µm in diameter. The number of neurospheres and their sizes were increased according to the amplitude and duration of BEC stimulation. Particularly, a magnitude of 8 µA/cm^2^ with 200 µs pulsed at 100 Hz exerted the most significant effect (3 days: ratio = 2.77±0.23, p<0.001; 5 days: ratio = 1.80±0.11, p<0.001) (Figure 4B). Although the BEC increased the number of large neurospheres compared to the control (Figure S1B) it did not significantly affect the total number of neurospheres including sub-100 µm sizes (Figure S1C). Electrical stimulation with relatively higher amplitudes (i.e., 16, 32 µA/cm^2^) or lower pulse durations (i.e., 50 µs) did not significantly affect proliferation (Figure 4).

### Electrical stimulation by BEC increased the neuronal population during differentiation of fetal NSCs

**Figure 5:**
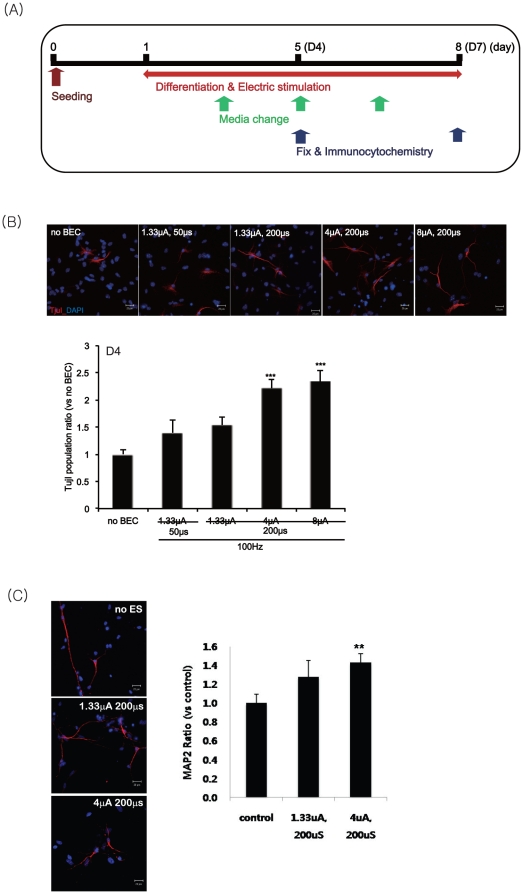
The effects of BEC on the neuronal differentiation during 4 days of differentiation. After 4 days of electrical stimulation at 100 Hz with a magnitude of 1.33, 4 or 8 µA/cm^2^ and duration of 50 or 200 µs, Tuj1 immunopositive neurons were counted for all differentiated cells. (A) Experimental scheme for the electrical stimulation during the differentiation. (B) Left panel: The differentiated NSCs were immunostained with anti-Tuj1 (red) antibodies and counterstained with DAPI (blue). Right panel: Counts of Tuj1 immunopositive neurons. DAPI nuclear staining is for the total numbers of neurons. Data represent mean ± S.E.M. of *n* = 15–20. ***, *p*≤0.001 by One-Way ANOVA: Tukey's HSD Post Hoc test. Scale bar = 20 µm.

We examined the effects of electrical stimulation by BEC on the differentiation of fetal NSCs into neuronal cells. We exposed the cells to a 100 Hz electrical stimulation with a magnitude of 1.33, 4 and 8 µA/cm^2^ with 50 and 200 µs pulse durations in a continuous manner according to the culture schedule shown in Figure 5A. Cells were fixed with 4% paraformaldehyde after 4 days and 7 days of exposure to biphasic electric currents (Figure 5A).

**Figure 6:**
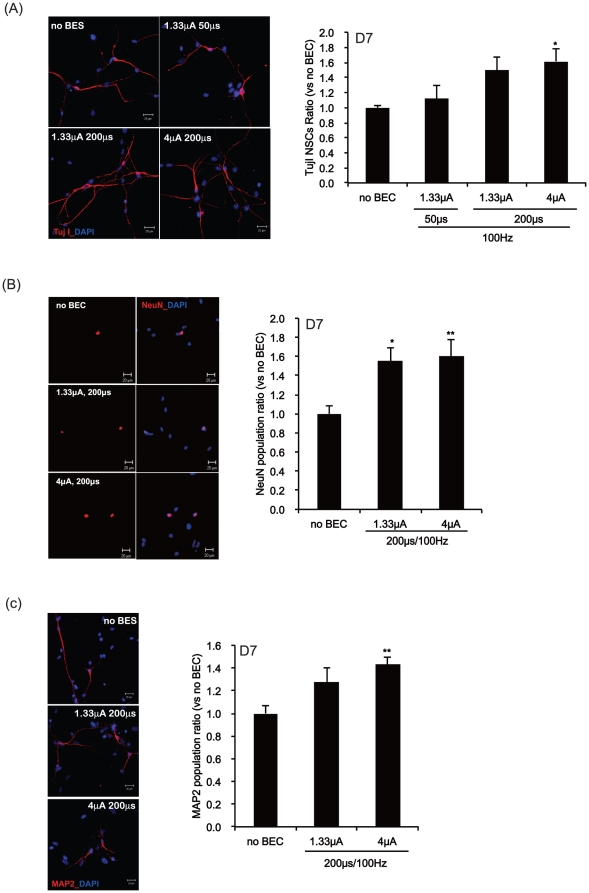
The effects of BEC on the neuronal populations during 7 days of differentiation. At day 7 after exposure to electrical stimulation at 100 Hz with a magnitude of 1.33 or 4 µA/cm^2^ and duration of 50 or 200 µs, Tuj1, NeuN or MAP2 immunopositive neurons were counted for all differentiated cells. (A–C) Left panel: The differentiated NSCs were immunostained with anti-Tuj1 (A; red), NeuN (B; red) or MAP2 (C; red) and counterstained with DAPI (blue). Right panel: Counts of Tuj1 (A), NeuN (B) or MAP2 (C) immunopositive neurons. DAPI nuclear staining is for the total numbers of neurons. Data represent mean ± S.E.M. of *n* = 25–30. *, *p*≤0.05, **, *p*≤0.01 by One-Way ANOVA: Tukey's HSD Post Hoc test. Scale bar = 20 µm.

Immunofluorescence study was performed using specific antibodies against βIII-tubulin (Tuj1), which labels neurons in early development as well as mature neurons [18], NeuN (neural nucleus marker), MAP2 (mature neuron marker), and glial fibrillary acidic protein (GFAP) to identify the differentiated level of astrocytic and neuronal populations. In differentiated NSCs treated with or without electrical stimulation, we compared the numbers of neurons stained with anti-Tuj1 and counterstained with DAPI for nuclear counting (Figure 5 & 6B). Tuj1 stains processes as well as cell bodies. Tuj1 positive cells were counted if a Tuj1 stained cell body included a DAPI stained nucleus to avoid double counting. To confirm whether the Tuj1 positive cells at 4 or 7 DIV were neurons, Fixed NSCs were, in addition, stained with NeuN (Figure S2A & Figure 6B) and MAP2 (Figure S2B & Figure 6C).

In differentiated NSCs at 4 DIV, the number of cells stained with the anti-Tuj1 antibody was increased by electrical stimulation compared with those of non-stimulated cells (Figure 5B). However, significant changes in the number of immunoreactive cells were not observed when stained with an anti-NeuN and MAP2 antibodies (Figure S2A & B). In differentiated NSCs at 7 DIV, the number of cells stained with the anti-Tuj1, NeuN or MAP2 antibodies was increased by electrical stimulation compared with that of non-stimulated cells (Figure 6). Especially, a 100 Hz electrical stimulation with a magnitude of 4 µA/cm^2^ for 200 µs in a continuous mode significantly increased the neuronal differentiation (Figure 5 & 6). In addition, we checked the proportions of cells positive for neuronal markers such as TujI, NeuN, or MAP2, and for glial marker, GFAP. We found that BEC increased the TujI-population to 17.0% (1.33 µA/cm^2^ for 200 µs) and 18.8% (4 µA/cm^2^ for 200 µs) compared with the control group (11.4%). The populations of NeuN were 9.4% in control; 14.6% in 1.33 µA/cm^2^ for 200 µs; 15.1% in 4 µA/cm^2^ for 200 µs. MAP2 positive portions were 9.9% in control; 13.3% in 1.33 µA/cm^2^ for 200 µs; 14.2% in 4 µA/cm^2^ for 200 µs. Glial marker, GFAP positive portion were 73.8%, 68.0%, and 65.0% respectively in control, 1.33 µA/cm^2^ for 200 µs, and 4 µA/cm^2^ for 200 µs (Figure S3C). Neuronal cell portions were increased by BEC but glial cell portion was slightly decreased.

## Discussion

The discovery of NSCs in the developing and adult brain that can generate neural tissue has raised new possibilities for repairing the nervous system [4]. The generation of synaptically active neurons from CNS stem cells has implications for the development of new cell therapies [19]. Although many investigators have been working to determine the key regulators of proliferation and the various pathways of differentiation, the fundamental mechanisms that regulate endogenous adult stem cells are poorly understood.

The criteria used to define neuronal differentiation from *in vitro* expanded stem cells has improved from the first experiments in which morphology and cell type-specific antigens for immature neurons were used ([8], [9]). The differentiation of stem cells to form functional neurons has been reported for cells derived from the substantia nigra [20]. In this case, nestin-positive precursors, expanded in bFGF (basic fibroblast growth factor), differentiated into tyrosine hydroxylase-positive neurons that secreted dopamine and were functional by behavioral tests when grafted into a rat model for Parkinson's disease [20]. Striatal stem cell-derived neuronal precursors preferentially differentiate into GABAergic neurons when exposed to BDNF (brain-derived neurotrophic factor) and other types of neurons after being exposed to insulin-like growth factor-I (IGF-I) [21]. Two neurotrophins, NT-3 and BDNF, influenced the differentiation of hippocampal stem cells into excitatory and inhibitory neurons [22]. However, stem cells can form neurons with functional synapses that respond to neurotrophic factors in similar ways to neurons derived directly from the developing brain [5].

One of the less explored non-chemical strategies for stem cell differentiation is the application of electromagnetic fields (EMF). Although there is enough evidence to believe that EMFs play an important role in cellular differentiation, the mechanism of their interaction with cells including effects on signal transduction pathways, changes in biosynthesis, induction of heat shock proteins (HSPs), and alteration on gene expression is not clear. According to a previous study, EMFs can modulate the initiation of the signal cascade pathways that regulate calcium fluxes [23]. Up- and down-regulation of calcium signaling is assumed to influence subsequences in mitogenesis [23]. EMFs can also induce free radical formation that regulates signal transduction pathway leading to control of gene expression and post translational modification of proteins ([24], [25]). EMFs can, in addition, alter cellular biosynthesis. It has been reported that biphasic electrical current (BEC), coupled with EMF, can up-regulate vascular endothelial growth factor (VEGF) leading to increased proliferation of osteoblasts [26]. The heat shock protein (HSP) activated by electrical stimulation has been shown to promote cellular differentiation. Transient expression of NeuroD2, one of neural basic helix-loop-helix, under the control of HSP activated by electrical stimulation converted mouse neuroblastoma cells into differentiated neurons [27]. The HSP mediated differentiation of human embryonal carcinoma cells into neuronal lineages has been also reported [28]. There is also evidence that EMFs can interact with nucleic acids. Exogenous EMFs may interfere with the endogenous EMFs and may alter gene expression ([29], [30]). Mouse embryonic stem cells will express cardiac lineage-promoting genes when exposed to extremely low frequencies of magnetic field [31].

Differentiation of stem cells into specific cell types will benefit from defined physicochemical properties. The combination of neurotrophic factors and EMFs can potentially enhance the proliferation and promote differentiation of NSCs in a more controlled manner. In regard to EMFs affecting stem cell biology, modulating factors include the wave-shape, frequency, amplitude, and pulse duration. It is supposed that EMFs are most effective when they coordinate with the natural rhythm of a reaction [32]. On the other hand, when the EMF does not coordinate with the natural rhythm, the proliferation and differentiation of NSCs might be inhibited.

This study focused on the proliferation and differentiation of neural stem cells under constant direct biphasic current using a biphasic electrical current (BEC) stimulator chip. The effect of electrical stimulation on the function of neural stem cells was analyzed *in vitro* for the first time in this study. We examined both the number of total NSCs and the number of neurospheres to determine the effect of the BEC on the proliferation of the neural stem cells (Figure 3, 4). We found that the number of large neurospheres (>100 µm) increased as well as the number of total neural stem cell counts. Self-renewing stem cells and progenitor cells a believed to comprise a neurosphere [33]. Progenitor cells have limited capacity to self-renew with increased passage numbers; therefore, neurospheres incorporating more progenitor cells will be smaller than those containing more self-renewing stem cells. After multiple days of culture, neurospheres that grew more than 100 µm in diameter were assessed and thought to contain more self-renewing stem cells than smaller sub-100 µm neurospheres (Figure 4). Thus, it was intriguing to find 8 µA/cm^2^ with 200 µs pulse durations of BEC (100 Hz) significantly increased the number of >100 µm neurospheres compared to the control while not essentially affecting the total number of neurospheres.

In addition, we showed that BECs with low amplitude and high pulse duration times (1.33, 4 µA/cm^2^; 200 µs pulse duration, 100 Hz) increased the numbers of NeuN, MAP2 and Tuj1 positive neurons from fetal neural stem cells cultured on laminin coated ITO glass (Figure 5, 6). At DIV-4, the population of Tuj1 positive cells in the electrically stimulated groups was significantly increased compared to the control, but the population of NeuN or MAP2 positive neurons did not significantly changed. At DIV-7, the Tuj1 positive neuronal population was also increased compared to the control (1.6-fold), which is similar with those cells positive for NeuN (1.6-fold) and MAP2 (1.4-fold). We checked differentiation populations after BEC treatment and found that NSCs preferentially differentiated into neuronal cells while glial cell production decreased (Figure S3). It appears long and continuous BEC stimulations can push neural stem cells to undergo neuronal differentiation.

In conclusion, our work suggests that (1) current densities greater than 1 µA/cm^2^ or less than 30 µA/cm^2^ for 200 µs at 100 Hz will positively affect the proliferation and the neural differentiation of fetal neural stem cells in our BEC culture system, (2) electrical stimulation at a calculated current density of 4 or 8 µA/cm^2^ for 200 µs at 100 Hz showed the greatest increase in proliferation, and (3) neural differentiation of fetal neural stem cells is significantly increased with 4 µA/cm^2^, 200 µs (100 Hz) electro-stimulation condition.

This *in vitro* study advances our understanding of how electric fields can alter fetal neural stem cell biology and can potentially contribute to the development of new stem cell therapies for various neurodegenerative diseases.

## Materials and Methods

### Preparation of fetal neural stem cells (fetal NSCs)

Fetal neural stem cells were derived from the cerebral cortices of the embryos (E13 days) of 6- to 7-week-old pregnant C57BL/6 mice (Japan SLC. Inc, Haruno Breeding Branch). Briefly, an anesthetized pregnant mouse was exposed to an overdose inhalation of 70–100% CO_2_ gas for euthanasia, and then the uterus was extracted into cold PBS. We collected embryos in cold PBS and then moved them into a filtered flask to administer euthanasia using an overdose inhalation of 70–100% CO_2_ gas. CO_2_ was chosen because it induced anesthesia and euthanasia rapidly and was highly effective. Next, we placed them in a fresh dish with PBS. Using curved, sharp forceps, the brains were collected from the embryos under a microscope. All animal experimental procedures were performed in accordance with 'the Guidelines of the Ethics Committee at Seoul National University' (SNU 091208-1).

### NSCs proliferation and differentiation

The generation of neurospheres derived from the neural stem cells of the cerebral cortices of mice was performed as described previously [9]. The NSCs were aggregated into neurospheres when subsequently plated and cultured in the proliferating media, DMEM/F12 (1∶1) (Gibco, NY). The medium was supplemented with 2 mM L-glutamine (Gibco), 0.6% glucose, 5 µM HEPES, 25 µg/ml insulin, 100 µg/ml apo-transferrin, 30 nM sodium selenite, 100 nM putrescine and 20 nM progesterone (all supplements purchased from Sigma, St. Louis) with growth factors of 10 ng/ml recombinant bFGF (Roche, Mannheim, Germany) and 20 ng/ml EGF (BD sciences, MA) at a density of 2×10^6^ cells in T75 flask (TPP, Switzerland) for 5 days. The neurospheres were then trypsinized (Trypsin EDTA, Gibco), triturated to a single-cell suspension, passaged in culture 3–5 times (P3-P5) with the media, and plated at a density of 5×10^4^ cells/well in the ITO glasses.

For the cell differentiation experiment, neurospheres were trypsinized (Trypsin EDTA, Gibco), triturated to a single-cell suspension, plated on the ITO glasses coated with Laminin (Roche), and then cultured in the differentiation media without growth factors (EGF and bFGF) for 4 or 7 days (4 DIV or 7 DIV). They were then assessed by immunocytochemistry.

### Biphasic electrical current stimulator chip (BECSC)

A biphasic current stimulator chip was fabricated using a 0.8-µm high-voltage complimentary metal-oxide semiconductor fabrication process at Austria Micro-Systems Corporation as described previously [34]. The chip was designed to generate continuous biphasic current pulses with diverse electrical parameters. The electrical parameters, such as amplitude, pulse width, and pulse rate, were programmable in the ranges of 2-1,024 µA (2 µA step, 512 levels), 25–775 µs (25 µs stepwise, 32 levels), and 5–3000 Hz (64 levels) respectively. An electrostatic discharge protector was built at all input and output pads of the chip to protect the circuit from external shock. An electrical short circuit between the reference electrode and stimulation electrode was also added in order to ensure safe electrical stimulation and discharge charges accumulated by the asymmetry between positive and negative phases. Figure 1A shows a circuit diagram of the stimulator chip. Biphasic current is generated by the complementary switching between ???, ??? and ???, ??? switches. The amplitude of the biphasic current is controlled stepwise by a 9-bit current digital to analog converter (DAC). The biphasic current pulses generated by the current stimulator were charge-balanced to avoid unintended chemical reactions by charge accumulation. Figure 1B shows a biphasic current stimulator where the stimulator chips, on/off power switch, and switches for parameter settings are installed. Figure 1C explains the current pulses.

### *In vitro* culture system on a biphasic electrical current stimulator chip

An *in vitro* culture system was designed in which dissociated NSCs could be cultivated and stimulated electrically. The culture system was made of Indium Tin Oxide (ITO) glass and Teflon jigs for biocompatibility with neuronal stem cells. The ITO glass with high electrical conductivity is so transparent that it is easy to observe cells through a microscope. Teflon is known to be excellent in chemical endurance and mechanical stability. Figure 2 shows a photo and a schematic drawing of the culture system. As shown in Figure 2B, two ITO glasses working as a stimulation electrode and a reference electrode were placed in parallel to deliver uniform current to the cells. The Teflon jigs formed culture chambers with the ITO glasses, and printed circuit boards (PCBs) were inserted for the connection between the ITO glass and the biphasic current stimulator. The *in vitro* culture system consisted of six identical chambers that were connected to the biphasic current stimulator (Figure 2A & 2B). Before and after assembly, all parts, including ITO glass and Teflon jig, went through a cleaning process using acetone, 70% ethanol, and de-ionized water for 15 min. After assembly, the culture system was also sterilized by plasma cleaner for 5 minutes.

### Antibodies

The primary antibodies used were as follows: rabbit polyclonal anti-glial fibrillary acidic protein (GFAP; 1∶500, Dako, CA); mouse monoclonal anti-βIII-tubulin (Tuj1; 1∶1000, Promega, CA); mouse anti-MAP2 monoclonal antibody (1∶500, Sigma); mouse anti-neuronal nuclei (NeuN; 1∶1000, Chemicon). The secondary antibodies used were as follows: Alexa fluor® 568 goat anti-mouse IgG (H+L) and Alexa fluor® 488 goat anti-rabbit IgG (H+L) (1∶1000, Molecular probes, Eugene, OR).

### Immunofluorescence and confocal microscopy

Cells grown on coated ITO glasses in a biphasic current stimulator chip were fixed for 20 min with 4% paraformaldehyde in PBS, pH 7.4, and rinsed three times with PBS. ITO glasses then were incubated for 60 min at room temperature (RT) or overnight at 4°C in PBS containing 4% NGS (normal goat serum, Vector laboratories, CA), 0.2% Triton X-100, 2% BSA (bovine serum albumin, Sigma), 2% FBS (fetal bovine serum, HyClone, Logan, UT) and the appropriate primary antibodies. After washing with PBS, the cells were reacted for 60 min at RT in the dark with the secondary antibody. ITO glasses were rinsed three times with PBS and mounted on glass slides with fluorescent mounting medium (DakoCytomation, CA).

After immunostaining, specimens were examined on a Zeiss LSM 510 confocal imaging system (Zeiss, Heidelberg, Germany) for immunofluorescence imaging. We also used this system for measuring the clonal diameter after immunostaining of the differentiating clones with neuronal markers.

### Cell toxicity Assay

The effect of BEC on cell toxicity was determined using LDH assay. The cells were examined 5 days during the BEC stimulation with various conditions. LDH activities in the medium were measured by a Cytotox 96 nonradioactive cytotoxicity assay kit (Promega) according to the manufacturer's instructions. Absorbance was measured at 490 nm with an ELISA reader (Molecular devices, CA). The results were expressed as percentages of peak LDH release obtained on addition of vehicle (0%), and complete cell lysis following addition of 10% Triton X-100 treatment (100%).

### Evaluation of the proliferation and neural differentiation of NSCs

We cultured NSCs in the form of single cell on coated biphasic current stimulator chip or in the sphere form on uncoated BEC chip under stimulating at 100 Hz with a magnitude of 4, 8, 16 or 32 µA/cm^2^ for 50 or 200 µs with the experimental scheme (Figure 3A). After 4 days of the electrical stimulation, both the numbers of total stem cells and the numbers of neurospheres were counted. Cell numbers were counted with trypan blue staining under the light microscopy. For the proliferation assay of the neurospheres, we attached a grid-inscribed scale (10 µm×10 µm) on the eyepiece in the light microscope (NIKON, Japan) and directly counted the spheres that were more than 100 µm in diameter. Data represent mean ± S.E.M. of *n* = 4–17 independent experiments.

For quantitative evaluation of neural differentiation with immunocytochemical data, the stem cells were stimulated at 100 Hz with a magnitude of 1.33, 4 or 8 µA/cm^2^ for 50 or 200 µs with the experimental scheme with the electrical stimulation during the differentiation (Figure 4A). The number of immune-reactive cells against the neuronal marker was counted and calculated on the basis of DAPI (Molecular Probes, Eugene, OR) stained total immunoreactive cells within the area. Data represent mean ± S.E.M. of *n* = 15–20 independent experiments.

### Statistical analysis

Data are expressed as the mean ± standard error of the mean (SEM) for triplicate or quadrant repeats with six independent samples (n>15) for the analyses of proliferation or differentiation. The results were statistically analyzed by the one-way ANOVA: Tukey's HSD Post Hoc test using PASW statistics (SPSS version 18). The difference was considered statistically significant for *, *p*≤0.05, **, *p*≤0.01, and ***, *p*≤0.001.

## Supporting Information

Figure S1 **The effects of BEC on the neuronal populations during 4 days of differentiation.** The stem cells were stimulated at 100 Hz with a magnitude of 4, 8, 16 or 32 µA/cm^2^ and duration of 50 or 200 µs. (A) A phase contrast micrograph of neurospheres after 4 days of electrical stimulation with grid of 100 µm. (B) Counts of neurospheres greater than 100 µm in diameter after 2 and 4 days of electrical stimulation. (C) Counts of total neurospheres of all sizes after 2 and 4 days of electrical stimulation. Data represent mean ± S.E.M. of *n* = 4–17. **, *p*≤0.01, ***, *p*≤0.001 by One-Way ANOVA: Tukey's HSD Post Hoc test.
(EPS)

Figure S2 **The effects of BEC on the neuronal populations during 4 days of differentiation.** After 4 days of exposure to electrical stimulation at 100 Hz with a magnitude of 1.33 µA/cm^2^ and duration of 50 or 200 µs, NeuN or MAP2 immunopositive neurons were counted for all differentiated cells. (A&B) Left panel: The differentiated NSCs were immunostained with anti-NeuN (A; red) or anti-MAP2 (B; red) and counterstained with DAPI (blue). Right panel: Counts of NeuN (A) or MAP2 (B) immunopositive neurons. DAPI nuclear staining is for the total numbers of neurons. Data represent mean ± S.E.M. of *n* = 10–15. Data was statistically analyzed by One-Way ANOVA: Tukey's HSD Post Hoc test. Scale bar = 20 µm.
(EPS)

Figure S3 **The effects of BEC on the neuronal populations during 7 days of differentiation.** The differentiated NSCs at DIV-7 were immunostained with anti-Tuj1 (A; red) or anti-MAP2 (B; red) and anti-GFAP (green) antibodies and counterstained with DAPI (blue). (C) The proportions for each cell marker of NeuN, MAP2, Tuj1, and GFAP.
(EPS)
