# Correction: Biphasic Electrical Currents Stimulation Promotes both Proliferation and Differentiation of Fetal Neural Stem Cells

**DOI:** 10.1371/annotation/99039a9d-1d1e-4059-93ea-f7f297c49d26

**Published:** 2011-05-10

**Authors:** Keun-A Chang, Jin Won Kim, Jeong a Kim, Sungeun Lee, Saeromi Kim, Won Hyuk Suh, Hye-Sun Kim, Sunghoon Kwon, Sung June Kim, Yoo-Hun Suh

The 4th author's name is misspelled. The correct spelling is: Sung Eun Lee. The correct citation is: Chang K-A, Kim JW, Kim Ja, Lee SE, Kim S, et al. (2011) Biphasic Electrical Currents Stimulation Promotes both Proliferation and Differentiation of Fetal Neural Stem Cells. PLoS ONE 6(4): e18738. doi:10.1371/journal.pone.0018738. 

